# Motor accidents, the first category of accidents and incidents in Sabzevar from 2001 to 2018: an epidemiological study

**Published:** 2019-08

**Authors:** Fateme Borzoee, Narjes Heshmati far, Raha Saleh Abadi, Mojgan Ansari

**Affiliations:** ^*a*^Department of Operating Room, School of Paramedics, Sabzevar University of Medical Sciences, Sabzevar, Iran.; ^*b*^Department of Nursing, School of Nursing, Sabzevar University of Medical Sciences, Sabzevar, Iran.

## Abstract

**Background:**

In the world, annually more than 5 million people die as a result of accidents, accounting for one tenth of all deaths. One of the most important strategies to deal with these cases is prevention, which necessitates recognizing the epidemiology of accidents and incidents. This study aims to investigate the epidemiology of accidents and incidents in Sabzevar in a period of 17 years.

**Methods:**

This is a descriptive cross-sectional study in which 125346 cases of accidents recorded from March 1, 2001 to March 13, 2018 were investigated. In this study, the information on the casualties referring to hospitals which had been collected thorough hospital registration forms was analyzed using software STATA version 11.

**Results:**

The total number of accidents recorded in Sabzevar County during 17 consecutive years has been 125,346. Most of the casualties were males (72.47%). Among all types of accidents, motor vehicle crashes accounted for most of the accidents with 29.53% of cases. Urban areas had the highest accident frequency rate with 68.43% of cases, compared to outdoors areas.

**Conclusions:**

According to the previous studies, Sabzevar is the second city in Iran with the highest number of motorcycles. It should be noted that these statistics are only related to the recorded accidents. Spreading the culture of riding bicycles as a means of transportation, equipping motorcyclists with helmets and other safety tools, a firmer approach toward violators and the enforcement of laws are more crucial today than they used to be.

**Keywords:**

Accidents, Epidemiology, Motor accidents

